# Can the Direct Medical Cost of Chronic Disease Be Transferred across Different Countries? Using Cost-of-Illness Studies on Type 2 Diabetes, Epilepsy and Schizophrenia as Examples

**DOI:** 10.1371/journal.pone.0147169

**Published:** 2016-01-27

**Authors:** Lan Gao, Hao Hu, Fei-Li Zhao, Shu-Chuen Li

**Affiliations:** 1 School of Biomedical Sciences & Pharmacy, The University of Newcastle, Callaghan, NSW, Australia; 2 Access and Public Affair, Pfizer Australia, West Ryde, NSW, Australia; University of Glasgow, UNITED KINGDOM

## Abstract

**Objectives:**

To systematically review cost of illness studies for schizophrenia (SC), epilepsy (EP) and type 2 diabetes mellitus (T_2_DM) and explore the transferability of direct medical cost across countries.

**Methods:**

A comprehensive literature search was performed to yield studies that estimated direct medical costs. A generalized linear model (GLM) with gamma distribution and log link was utilized to explore the variation in costs that accounted by the included factors. Both parametric (Random-effects model) and non-parametric (Boot-strapping) meta-analyses were performed to pool the converted raw cost data (expressed as percentage of GDP/capita of the country where the study was conducted).

**Results:**

In total, 93 articles were included (40 studies were for T_2_DM, 34 studies for EP and 19 studies for SC). Significant variances were detected inter- and intra-disease classes for the direct medical costs. Multivariate analysis identified that GDP/capita (p<0.05) was a significant factor contributing to the large variance in the cost results. Bootstrapping meta-analysis generated more conservative estimations with slightly wider 95% confidence intervals (CI) than the parametric meta-analysis, yielding a mean (95%CI) of 16.43% (11.32, 21.54) for T_2_DM, 36.17% (22.34, 50.00) for SC and 10.49% (7.86, 13.41) for EP.

**Conclusions:**

Converting the raw cost data into percentage of GDP/capita of individual country was demonstrated to be a feasible approach to transfer the direct medical cost across countries. The approach from our study to obtain an estimated direct cost value along with the size of specific disease population from each jurisdiction could be used for a quick check on the economic burden of particular disease for countries without such data.

## Introduction

With greater emphasis on rational distribution of healthcare resources, the availability of economic evaluation has increased dramatically in recent decades. However, there is still a paucity of local economic evaluation to assist many jurisdictions that are attempting to make reimbursement decision. In interpreting these results from other jurisdictions, decision-makers need to form a view on whether the results are transferable into their own settings. In health economics, transferability refers to adopt the results from foreign studies to one particular country with/without certain adaptations, while does not need a new national study for that medical technology. However, the sometimes huge difference in health care systems, such as the variation in clinical practice patterns (e.g. the difference in hospitalization rate), in relative prices, and the availability of the alternative treatments, hinders such attempt [1–3].

To appreciate the difficulties encountered by the decision makers in adopting economic evaluation from other jurisdiction, we need to examine what type of information needs to be deciphered. When considering the decision about cost-effectiveness of any new health treatment or technology, the obvious answer is whether the incremental cost-effectiveness ratio (ICER) is applicable locally. An ICER comprises of a cost component and an effect component. The decision makers will need to evaluate whether these two components individually and collectively from a published study can be applied in their own jurisdictions. Management of various diseases or disorders is fairly standardized internationally and thus the effect component may pose fewer problems. Often times, it is the cost component that is creating more concern when trying to transfer across jurisdictions.

From the societal perspective, the economic cost of an illness has three important components: direct costs, indirect costs and intangible costs. Due to various unresolved technical and theoretical issues, in most economic evaluation, intangible cost is usually not reported or included. For the two remaining components: indirect cost has previously been demonstrated to be transferrable when local data were unavailable by our research group [4]; while the issue of whether the direct cost component is transferrable still remains unresolved. In order to answer this question, we would need to gather information from Cost-of-illness studies performed internationally.

Cost of illness (COI) is one form of economic studies and it is often used to estimate the economic burden of a particular disease. Such knowledge can help policy makers to set priority in health care manpower planning, resource allocation and prevention policy [5–8]. Most importantly, COI studies are able to show whether new treatments could be valuable in reducing the burden of a specific disease. It can also provide important information for cost-effectiveness and cost-benefit analysis by providing the cost estimation in these analyses. As a tool, COI study is a better capture of cost information for treating a disease compared to cost-effectiveness or cost-utility analysis. In practice, cost-effectiveness or cost-utility analysis only concerns the direct medical cost estimates for different health states or events that are tracked over time in a model, whereas COI studies typically estimate all costs related to that disease over a year (prevalence-based) or even over a life-time (incidence-based). It would provide more information for decision-makers in terms of costs. Ideally, if COI could be conducted for various diseases and disorders locally, the information could be utilized by the decision makers immediately. Unfortunately, this is not the case and for most diseases, local COI study is not available, especially for a lot of developing countries.

Aside from the substantial resources required to carry out COI study, even if it is feasible for each jurisdiction to conduct a COI study to gauge the economic burden of a disease, there will be substantial time delay affecting funding recommendations for new programs [2].A reference value for a quick check on the cost of a particular disease would really be helpful for decision-makers needing to make a decision within a very short time frame. To date, the vast majority of studies performed were to explore the transferability of cost-effectiveness studies. A literature review indicated that the factor most frequently cited as generating variability in economic results between locations was the unit costs associated with particular resources, e.g. the absolute or relative prices of resources [9]. Accordingly, several statistical methods have been proposed to tackle the problem of distinguishing location-specific costs. Studies have been performed using regression model to look at variability in the cost among six European countries based on data from a randomized trial. Using country-specific unit costs, individual patients’ costs within each country were summated. However, it was concluded that it was difficult to pool the cost data from the six countries due to significant differences among them and the authors also emphasized the importance of separating resource use from cost rather than simply reporting and analysing total costs[10]. A similar method using an adjusted resource quantity to a “typical treatment” pattern of the target country while substituting the unit prices of the target country was also proposed [11]. Other researchers suggested running regression analysis on cost components where variation between locations is evident, to assess the degree to which variation can be explained by various factors[12]. Nevertheless, none of the aforementioned methods can provide a reference value for decision-maker with limited or no information. Approaching from another angle, confirmation of the transferability of COI study across jurisdiction would contribute significantly to resolving the issue of transferability of cost-effectiveness results.

However, COI studies varied substantially in terms of perspective, cost component, source of data, calculating methods, the time frame of measurement and therefore the final result [13]. Due to these heterogeneities, studies have rarely been conducted to explore transferability of COI studies from country to country. So far, only one recent study has been carried out to investigate the transferability of COI study (indirect cost due to chronic diseases)[4]. In this study, after transforming the monetary value into percentage of GDP per capita, the large variation in indirect cost became much narrower across the studies, thus providing a meaningful reference range to estimate indirect cost. While this may solve the issue of transferability of indirect cost, direct cost especially the direct medical cost is still an irreplaceable cost component and the primary concern from the healthcare provider’s/third party perspective. Therefore, transferring direct medical costs across countries (or at least providing some meaningful interpretation) could considerably aid the healthcare decision-making, particularly for countries with limited resource and research output. Hence, in our current study, three chronic diseases were selected—type 2 diabetes mellitus, epilepsy and schizophrenia to review the direct medical cost via meta-analysis with the aim of identifying the primary determinants for this cost component, and the transferability across countries.

## Methods

### Search strategies and selection criterion

Electronic databases including Medline, Embase, Cochrane Library and EconLit were searched from inception to 15^th^ April, 2013. The following terms were used to locate the relevant literatures: cost of illness, cost, burden of illness, cost analysis; and then followed by a disease filter with ‘type 2 diabetes mellitus’ (T_2_DM), ‘epilepsy’ (EP), or ‘schizophrenia’ (SC) as key words separately. For the different searches, phrases were conjoint by Boolean operators “AND” and “OR” (The electronic search strategy is provided Supplementary document). In addition, manual search was also performed of references from identified literatures. Since the present study intended to adopt the healthcare system perspective, direct non-medical cost was beyond the scope of our research.

Inclusion criteria (studies should satisfy all the following criteria):

1. Patients were diagnosed with T_2_DM, EP or SC.
2. It was original research.
3. Direct medical cost should at least encompass three of the following cost components: hospitalisation, outpatient, medication, examination/laboratory test/procedure.
4. Direct medical cost per patient annually was reported or could be calculated in monetary term.
5. Estimation was based on incidence or prevalence of the disease of interest.
6. Studies utilized bottom-up, top-down, econometric or modelling method to calculate the direct medical cost.

At first, titles and abstracts of all the yielded articles were used to screen out irrelevant literatures. Then, full-text of the remaining ones were reviewed and checked on the eligibility for inclusion.

### Literature synthesis

A standardized collection form was used to extract data from eligible studies. Extracted data included year of publication/conduction, country/region of study, data source, calculation method, incidence or prevalence-based, retrospective or prospective, number of patients, demographic characteristics of patients, monetary value of direct medical cost and each component’s cost. Specific year of currency exchange rate was used to convert the reported cost into US dollars, and adjusted to 2011 value based on US consumer price index (CPI). If the year of study conduction was not explicitly stated, the publishing year was adopted. The Gross Domestic Product (GDP) per capita in 2011 for the included countries was obtained from the World Bank [14]. The direct medical cost was expressed as percentage of the particular country’s GDP/capita in 2011.

### Data analysis

#### Multivariate analysis

In order to identify the factors that contributed to the large variation in the direct medical cost, generalized linear model with gamma distribution and log link was employed to examine the effects of categorical and continuous variables on the raw direct medical cost given the skewness of the cost data. In this process, direct medical cost was selected as the dependent variable and the methodological (e.g. data source [published literature, database, survey], calculation method [bottom-up, top-down, modelling], were dummy coded where applicable) and socio-demographical variables (GDP/capita) were treated as fixed factors in this model.

#### Meta-analysis

For the meta-analysis, direct medical cost expressed as percentage of GDP per capita was input as the individual cost estimate. Both parametric (Random effects) [15] and Non-parametric (Bootstrapping) methods [16] were adopted to compute the mean and 95% confidence intervals (CI) of the weighted cost estimate [4]. Since random-effects model accounts for the random effects variance, which represents the variability across the population effects, the biggest difference between fixed-effects and random-effects models is the significance levels (effects that were significant under a fixed-effects model may no longer be significant) and confidence intervals (confidence intervals will get bigger). Furthermore, if sample size is highly related to cost estimate, then the mean cost estimate will differ between the two models. So the random-effects model will generate more conservative result comparing to fixed-effects model and the fixed-effects model was not adopted in our analysis. For the bootstrapping analysis, a random number of studies with replacement were chosen and then a weighted mean cost estimate was calculated in the bootstrapping method. This process was repeated for another 1999 times, the output values were rank-ordered sequentially, and the lowest and highest 2.5% values chosen as the bootstrap confidence limits. As majority of the included studies did not present the variance of annual cost probably due to highly skewed data, the cost estimate was weighted by the sample size of each study instead of variance in parametric meta-analysis [15]. In order to further compare the raw cost and converted percentage of GDP/Capita, the within- and between-country variations were calculated using country of origin as the clustering factor before and after the conversion for the total cost. Moreover, the intraclass correlation coefficient (ICC or *ρ*) was estimated using the between-country and within-country variations calculated above.

For missing values on sample size in the studies, they were replaced by the average of remaining studies for that particular variable within a disease subgroup for the meta-analysis. In addition, in order to avoid putting unbalanced weight on study with extreme sample size, sample size for studies with greater than 10,000 subjects were replaced with 10,000. Therefore, sample sizes of 16, 7 and 4 studies from T_2_DM, SC and EP disease groups were truncated through this approach, respectively. Data were analyzed using SPSS 20.0 (SPSS Inc. Chicago, IL, USA) and Microsoft Excel. A detailed protocol is presented in supplementary document.

## Results

### Identify the relevant studies

**Fig 1:**
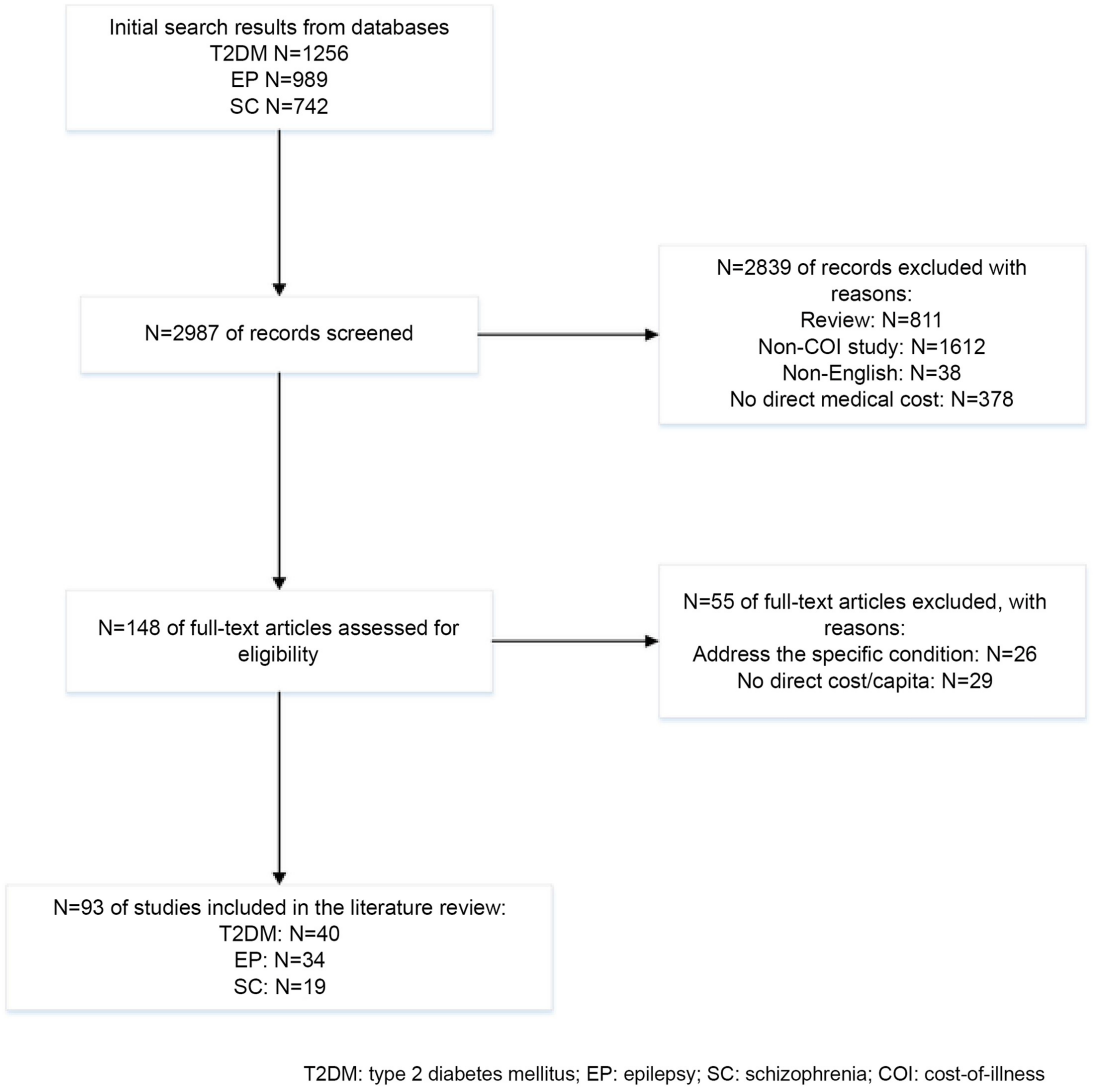
Selection process of the eligible studies.

The initial electronic database search yielded 2987 papers, but 2839 were excluded based on screening of titles and abstracts. Then the remaining 148 articles were retrieved for full-text review to further examine the eligibility. However, 55 studies were not included as they only addressed one specific aspect of the disease or per capita direct cost was not provided (or could not be calculated). After this culling process, 93 studies finally met the inclusion criteria for the study. There were 40 studies for T_2_DM [17–56], 34 studies for EP,[57–90] and 19 studies for SC [91–109]. The detailed culling process was shown in Fig 1.

### Description of the included studies

The characteristics of each included study and a summary of all the included studies based on the disease group were presented in Table 1 and S1 Table. Generally, all studies addressed cost components for outpatient care, medications, examination and/or hospitalization, though not all presented those components along with the total cost.

**Table 1:** Characteristics of literature synthesis.

Disease	Time range	Countries	Survey design (%)	Prevalence-based study (%)	Bottom-up design (%)	Prospective study (%)	Inpatient cost reported (%)	Outpatient cost reported (%)	Medication cost reported (%)	Tests cost reported (%)	Characteristics of subjects (%)	Range of direct medical costs (USD)
DM	1992–2012	23	23 (47.9)	48(100)	23 (47.9)	1 (2.1)	32 (66.7)	33 (68.7)	35 (72.9)	9 (18.7)	25 (52.1)	164–20568
EP	1990–2011	15	20 (54.1)	30 (81.1)	23 (62.2)	7 (18.9)	34 (91.9)	33 (89.2)	35 (94.6)	32(86.5)	23 (62.2)	2–13787
SC	1990–2008	16	13(61.9)	20 (95.2)	14 (66.7)	5 (23.8)	14 (66.7)	13 (61.9)	13 (61.9)	0 (0)	10 (52.4)	101–33627

Studies with multiple estimations were deemed as different studies.

DM: diabetes mellitus; SC: schizophrenia; EP: epilepsy.

Several studies presented different estimations for various patient subgroups, thus each was treated as an independent study and the results were shown separately[27, 29, 30, 39, 45, 46, 63, 67, 78, 108].

For T_2_DM, there were 48 studies (including six studies that provided cost values for different subgroups, with two of these studies estimated the costs for three subgroups) (15, 17, 18, 27, 33, 34) performed in 23 countries from 1992 to 2012 (S1 Table). All the studies estimated direct medical cost based on prevalence. Among these, 23 (47.9%) studies utilized the survey approach and published literatures/expert opinions to generate the data. 19 studies (39.6%) used the top-down method by accessing the national statistics or regional databases to calculate the cost. Only one study (2.3%) adopted the prospective perspective to collect data. In terms of cost components, more than 65% of the studies reported individual cost for hospitalization, outpatient care and medications, whereas only 9 (18.8%) studies presented the costs for examinations explicitly. As a result, the estimated direct medical cost ranged from $104 (India) to $ 20568 (USA), showing a huge range from developing to developed countries.

In EP group, 37 studies (three studies estimated the costs using different methods) (51, 55, 66) were conducted in 15 countries between 1990 and 2011. A majority of studies (20 studies, 54.1%) using survey data as their data sources. 30 and 7 studies computed the cost based on prevalence and incidence respectively. Bottom-up (23 studies) and retrospective (30 studies) data collection approaches were still being more frequently used than top-down and prospective methods. Availability of cost for hospitalization, outpatient care, medication, examination were the greatest for this disease group, with more than 80% of the studies reporting each of the cost components. Finally, the average direct medical cost varied from $2 (Burundi) to $13787 (USA) per annum.

For SC group, 21 studies (the Spanish study was divided into three studies according to different prevalence estimations (96)) were carried out in 16 countries from 1990–2008. Again, greater number of studies used survey data and all the studies estimated direct medical cost by the prevalence-based approach. Retrospective data collection method was used in 5 studies (23.8%).More than 60% of studies explicitly reported the cost for inpatient, outpatient care and medication costs, but none presented the costs for examination. The reported annual direct medical costs for epilepsy patient amounted from $101 (Nigeria) to $33627 (England).

### Contributor to the variation in direct medical cost

With direct medical cost as dependent variable, categorized, year of publication, GDP/capita, data source, calculation method, and time perspective were entered into the model as fixed factors. As a result, GDP/capita contributed considerably to all three models for T2DM, EP, and SC (Tables 2–4). Due to limited number of studies in each model, other variables that significantly contributed to models were inconsistent across three diseases, which disabled us to draw any reliable conclusion.

**Table 2:** Results from generalized linear model analysis (Type 2 Diabetes Mellitus).

Tests of Model Effects			
Source	Type III		
	Wald Chi-Square	df	Sig.
(Intercept)	870.654	1	<0.0001
Publication period	3.459	2	0.177
Methods of cost computation	1.983	2	0.371
Data source	2.380	2	0.304
Longitudinal study	13.217	1	<0.0001
GDP/Capita	508.481	22	<0.0001

Number of study: 48.

**Table 3:** Results from generalized linear model analysis (Epilepsy).

Tests of Model Effects			
Source	Type III		
	Wald Chi-Square	df	Sig.
(Intercept)	1006.374	1	<0.0001
Publication period	7.815	2	0.020
Methods of cost computation	10.541	2	0.005
Data source	32.857	5	<0.0001
Longitudinal study	0.974	1	0.324
GDP/Capita	658.594	15	<0.0001

Number of study: 37.

**Table 4:** Results from generalized linear model analysis (Schizophrenia).

Tests of Model Effects			
Source	Type III		
	Wald Chi-Square	df	Sig.
(Intercept)	28104.449	1	<0.0001
Publication period	26.147	2	<0.0001
Methods of cost computation	216.785	1	<0.0001
Data source	211.113	1	<0.0001
Longitudinal study	125.762	1	<0.0001
GDP/Capita	13632.364	10	<0.0001

Number of study: 21.

### Meta-analysis

In general, direct cost expressed as a percentage of GDP/capita showed substantially narrower 95% CI than the cost expressed in US dollar values. The meta-analysis results based on parametric and non-parametric methods did not differ substantially from each other for all disease groups. Particularly, means and 95% CIs of the synthesized results derived from random-effects model and bootstrapping estimation were similar. The converted direct medical costs for DM, SC and EP were ranged between 10% and 23%, 19% and 53%, 7% and 13% of GDP/capita according to different estimation methods respectively, on contrast, the interquartile ranges of raw costs were between $906 and $5879, $3456 and $23310, $1586 and $4848, separately. Still, SC incurred higher direct medical cost than the other two diseases (Table 5). The between-country variation demonstrated that the GDP/Capita adjusted total cost had substantially lower between country heterogeneities than the raw total cost (2.45 vs. 0.28) (Table 6).

**Table 5:** Raw cost and meta-analysis of direct medical costs.^†^

	Raw cost	Random effect	Bootstrapping
	Median (IGR)	Mean (95% CI)	Mean (95% CI)
Diabetes Mellitus	$3186.50	16.4666^‡^	16.4283
	(906, 5878.75)	(10.2033, 22.7299)	(11.3242, 21.5425)
Schizophrenia	$8332.00	36.2242^&^	36.1686
	(3455.50, 23310.00)	(19.1496, 53.2988)	(22.3380, 49.9991)
Epilepsy	$2660.0	10.3798^§^	10.4916
	(1585.50, 4847.50)	(7.4273, 13.3322)	(7.8604, 13.41229)

IGR: interquartile range.

^†^ direct medical costs were expressed as percentage accounted for the GDP/Capita.

^‡^Q = 30.77, I^2^ = 0

^&^Q = 13.38, I^2^ = 0

^§^ Q = 28.49, I^2^ = 0.

**Table 6:** Comparison of between and within country variation in total cost.

	Between-country variation	Within-country variation	ICC (*ρ)*
Raw cost data	2.454	0.394	0.86
GDP/Capita adjusted cost data	0.279	0.428	0.39

ICC: intracluster correlation coefficient.

## Discussion

Our study systematically reviewed cost of illness studies addressing the direct medical cost for type 2 diabetes mellitus, epilepsy and schizophrenia and explored the determinants for the variation in this cost component. Unsurprisingly, the direct medical costs varied substantially across different countries. The discrepancy existed among the developed countries (and sometimes even studies for the same country), would render meaningless any attempt for the meta-analysis of COI studies if the raw data were used. Furthermore, it was reported that resource use is considered of low transferability in a review of pharmacoeconomic guidelines in regard to transferability of economic data, obviously, it is beyond argument. However, converting the raw cost into a new currency (percentage of GDP/Capita) is different from those discussed in the review, which may aid to resolve this problem[110]. After converting the raw value of direct medical cost into percentage of local GDP/capita, the variation of the direct medical cost reduced markedly, which was supported by the reduced between-country variation. The ICC of the raw cost data was greater than the converted cost as the GDP/capita is a differentiating factor rather than a correlation factor. The proposed method is able to reduce the between-country variance while does not alter the within country variance. Ideally, each study should be weighted by the inverse variance from that study in the meta-analysis. However, a large number of the studies did not report the 95% confidence interval or the standard deviance, which makes using such weighting method unrealistic. As an alternative, the sample size was adopted to approximately weight each study in our meta-analysis. In addition, non-parametric meta-analysis method (boot-strapping meta-analysis) was performed to further test the robustness of our results.

Via these two approaches, the mean and 95% CI of the direct medical cost of each included disease was estimated as the percentage of local GDP/capita, and this substantially reduced the ranges of variations in the direct medical costs across countries. Hence, our study provided a new potential approach to transfer the direct medical cost across countries. The synthesis of direct medical cost of commonly occurring chronic diseases along with the patient population size of that particular disease could be employed to provide a quick check of the burden of the disease when such information is not readily available. For example, with an extremely limited healthcare budget, the policy-makers in China need to decide whether to reimburse an antiepileptic drug or an anti-schizophrenia drug provided that these two drugs have very similar Incremental Cost-effectiveness Ratio values. This would be difficult if the direct treatment costs for the two diseases were unavailable. In this case, using the approach from our present study of utilizing information from other jurisdictions together with the sizes of these two patient populations can help policy-makers to set the priority and make the reimbursement choice. Additionally, this information can also provide an estimate of the quantum of changes in direct costs of any claimed benefits from a new drug or intervention at the population level.

There are several rationales for using the GDP/capita to adjust the direct medical cost. Firstly, as demonstrated in the multivariable analysis, GDP/capita was positively associated with direct medical cost. It is also well acknowledged that the difference in health care systems is an independent factor causing the variability in the direct medical cost (as countries may vary in terms of the types and magnitude of health care resources, programs, or services that are available). Furthermore, the development of health care system is always determined by the economic status of the individual country. For instance, the highest cost of each disease was always generated by the developed country from our study (USA and England), as absolute and relative prices in healthcare, practice variation and technology availability all have direct impact on either direct medical cost or cost of care[111]. Since GDP/capita is a widely accepted index to measure the economic performance of a country, adjusting the direct medical cost by GDP/capita could account for the variability caused by health care system to a large extent. The results from our present study supported the feasibility of such conversion. Certainly, other factors associated with cost variability such as characteristics of patients (demographics, types of insurance coverage), disease (epidemiology, disease mortality), provider (clinical practice, guidelines) and methodologies (costing method, study perspective) also contribute to the difference in the medical cost estimation.[2] Nonetheless, the possible impact of these factors may be accounted for by the 95% CI of the values. Therefore, based on the assumption of the economic status of each country and the health care system are the major contributors to the large variation in direct medical costs across jurisdictions, it is feasible to synthesize the direct medical cost expressed as the percentage of GDP/capita to establish the bounds of each chronic disease to provide informative data to country/jurisdiction without such information. The estimates (with its 95% CI) would also serve as a comparator of assessing service efficiency for country/jurisdiction with such data.

A previous study from our research group utilized the same approach to transform the indirect cost including the mortality and caregiver’s costs into percentage of GDP/capita, and demonstrated the feasibility of the approach in allowing transferability of indirect cost data across jurisdictions [4]. Our current study broadened the potential usefulness of such approach to direct medical cost. The transferring of both direct and indirect medical costs across jurisdictions can provide a more comprehensive view on economic burden of a particular disease when making any health resource allocation decision in lieu of local information.

Practically, the usefulness of our study would largely be for developing countries where economic evaluation study is seldom conducted. With the increasing constraints in health budget and the ever-escalating demands internationally, it is necessary to adopt economic evaluation in supporting the health care decision-making. The missing information on the economic burden of chronic disease would definitely hamper the rational distribution of health resource, particularly in developing countries where resource is more limited. Therefore, the approach from our study could offer a mean of obtaining this reference information to assist in decision making. Even in countries with these data, the mean value from pooled studies could be utilized for benchmarking comparisons.

Nevertheless, the predictive capability of the constructed mean and range in predicting direct medical cost of DM, EP and SC in a country not included in our study needs to be validated by more studies. For instance, it would be desirable to use our method to predict the direct medical cost in a jurisdiction prior to conducting a study to directly estimate the direct medical cost. Researchers should apply caution to directly utilize the 95% CI from the bootstrap meta-analysis to construct a 95% CI for a target country not yet studies in the presented meta-analysis as the CI obtained by bootstrap method is a confidence interval for the mean rather than that for an individual country.

Inherently, several limitations are worth mentioning. First of all, due to the difference in the economic status affecting resource availability, patients with those chronic diseases in developing countries may not be managed optimally according to the international guidance. However, expressing direct medical cost as percentage of GDP/capita might minimize such impact to a certain extent. Second, the heterogeneities in the methodology of each study also introduced another uncertainty. To compensate, we tried to integrate the methodological variables into the multivariate analysis model. From the result, data source, year of study conduction, time perspective (retrospective or prospective), and disease definition (incidence or prevalence-based) were not significant contributors to the model. Third, only three chronic diseases were investigated in the present study, further study utilizing the same method on other diseases would enhance the generalizability and validity of our approach. It is planned that with the emerging of more COI studies reporting on the included diseases, an external validation of the proposed method could be performed via reconverting the percentage of GDP/capita to the monetary form (percentage of GDP/Capita× GDP/Capita from particular country of interest), to see if the direct medical cost from a new study could fall within the range constructed in the presented study.

## Conclusions

The direct medical cost for schizophrenia, type 2 diabetes mellitus and epilepsy varied substantially across different countries and even for the same country. However, when converted the raw cost data into percentages of GDP/capital of each country, the variance in the cost became much narrower. Pooling the converted raw cost data can be of help to construct a reference range for other countries without such data. The mean percentage of GDP/capita estimate can be converted back to the monetary value of the jurisdiction of the decision maker. When combined with the size of patient population in a jurisdiction, it can provide a quick check on the economic burden of a particular disease.

## Supporting Information

S1 PRISMA Checklist (PDF)

S1 Protocol (DOCX)

S1 Table Characteristics of included studies (2011 USD). (DOCX)
