# Supplementary material for: Can the Direct Medical Cost of Chronic Disease Be Transferred across Different Countries? Using Cost-of-Illness Studies on Type 2 Diabetes, Epilepsy and Schizophrenia as Examples
Source: PLoS One. 2016 Jan 27;11(1):e0147169. doi: 10.1371/journal.pone.0147169 (PMC4731392; doi:10.1371/journal.pone.0147169)
Supplement: S1 Protocol — (DOCX) [file pone.0147169.s002.docx]

**S1_Protocol**

**Systematic review and meta-analysis of cost of illness studies for epilepsy, schizophrenia and type 2 diabetes mellitus—study protocol**

**Introduction**

Rationale

Cost of illness (COI) is one form of economic studies and it is often used to estimate the economic burden of a particular disease. Such knowledge can help policy makers to set priority in health care manpower planning, resource allocation and prevention policy. Most importantly, COI studies are able to show whether new treatments could be valuable in reducing the burden of a specific disease. Additionally, COI studies can also provide important information for cost-effectiveness and cost-benefit analysis by providing the cost estimation in these analyses. As a tool, COI study is a better capture of cost information for treating a disease compared to cost-effectiveness or cost-utility analysis. From the societal perspective, the economic cost of an illness has three important components, namely, direct costs, indirect costs and intangible costs. However, due to various unresolved technical and theoretical issues, in most economic evaluation, intangible cost is usually not reported or included. For the two remaining components, our research group has previously demonstrated that indirect cost estimated from other jurisdictions may be transferrable when local data were unavailable ([Zhao, Xie et al. 2013](#_ENREF_4)).

Objectives

The main objective is to systematically review the existing cost of illness studies reporting on the direct medical cost. Three chronic diseases were selected—type 2 diabetes mellitus (T2DM), epilepsy (EP) and schizophrenia (SC) to meta-analyse the direct medical cost, with the aim of identifying the primary determinants for this cost component, and the transferability across countries. These three diseases were selected to correspond to the previous study conducted by our research group that investigating the transferability of indirect cost across jurisdictions. It should be noted that these three diseases are just examples to exemplify the feasibility of the proposed method to transfer cost data across different nations. The method can be applied to other diseases if the method is demonstrated to be valid.

**Methods**

Eligibility criteria

1. Patients were diagnosed with T_2_DM, EP or SC.
2. It was original research.
3. Direct medical cost should at least encompass three of the following cost components: hospitalisation, outpatient, medication, examination/laboratory test/procedure.
4. Direct medical cost per patient annually was reported or could be calculated in monetary term.
5. Estimation was based on incidence or prevalence of the disease of interest.
6. Studies utilized bottom-up, top-down, econometric or modelling method to calculate the direct medical cost.

Information source

Electronic databases including Medline, Embase, Cochrane Library and EconLit were the only information source, which were searched from inception until April, 2013.

Search strategy

Electronic search strategy [Medline, 15^th^ April, 2013]

1. cost of illness/
2. cost analysis/
3. health expenditures/
4. hospital costs/
5. ‘burden of illness’
6. 1 OR 2 OR 3 OR 4 OR 5
7. Diabetes mellitus, Type 2/
8. Schizophrenia/
9. Epilepsy/
10. 6 AND 7
11. 6 AND 8
12. 6 AND 9
13. 10 OR 11 OR 12

Study records

*Data management, selection process and data collection process*

A standardized collection form was used to extract data from eligible studies. Two reviewers (LG and FLZ) were responsible for data extraction independently. Only agreed data by two reviewers were extracted for data analysis. Any discrepancies were resolved by the third reviewer (SCL).

*Data items*

Year of publication/conduction, country/region of study, data source, calculation method, incidence or prevalence-based, retrospective or prospective, number of patients, demographic characteristics of patients, monetary value of direct medical cost and each component’s cost.

*Outcomes*

The primary outcome was the percentage of direct medical cost accounting for the particular country’s GDP/capita in 2011. It was converted via three steps:

1. Specific year of currency exchange rate was used to convert the reported cost in each study into US dollars. If the year of study conduction was not explicitly stated, the publishing year was adopted.
2. Then it was adjusted to 2011 value based on US consumer price index (CPI). (http://www.usinflationcalculator.com/)
3. The Gross Domestic Product (GDP) per capita in 2011 for the included countries was obtained from the World Bank . The primary outcome was calculated via the following formula:

$$Percentage form of direct medical cost\left( \% \right)=\frac{Direct medical cost(USD)}{\frac{GDP}{capita}(USD)}$$

The rationales for the aforementioned conversion are that: Firstly, as demonstrated in the multivariable analysis, GDP/capita was positively associated with direct medical cost. It is also well acknowledged that the difference in health care systems is an independent factor causing the variability in the direct medical cost (as countries may vary in terms of the types and magnitude of health care resources, programs, or services that are available). Furthermore, the development of health care system is always determined by the economic status of the individual country. Since GDP/capita is a widely accepted index to measure the economic performance of a country, adjusting the direct medical cost by GDP/capita could account for the variability caused by health care system to a large extent. Based on the assumption of the economic status of each country and the health care system are the major contributors to the large variation in direct medical costs across jurisdictions, it is feasible to synthesize the direct medical cost expressed as the percentage of GDP/capita to establish the bounds of each chronic disease to provide informative data to country/jurisdiction without such information.

Secondly, a previous study from our research group utilized the same approach to transform the indirect cost including the mortality and caregiver’s costs into percentage of GDP/capita, and demonstrated the feasibility of the approach in allowing transferability of indirect cost data across jurisdictions ([Zhao, Xie et al. 2013](#_ENREF_4)). Our current study broadened the potential usefulness of such approach to direct medical cost. The transferring of both direct and indirect medical costs across jurisdictions can provide a more comprehensive view on economic burden of a particular disease when making any health resource allocation decision in lieu of local information.

The secondary outcomes includes costs of hospitalization, outpatient care, medications, and tests/examinations.

*Risk of bias in individual studies*

As the potential studies were not randomized controlled trials or cohort studies, it was not applicable to assess the risk of bias in each study.

*Data analysis*

Multivariate analysis

In order to identify the factors that account for the variation across different COI studies, a multiple linear regression model was adopted. In the first model, all the studies (regardless of disease group) entered into the model. Raw direct medical cost was selected as the dependent variable (given the skewness of cost data, the logarithm form was used in the model); all the other characteristic variables extracted from individual studies that were categorized in advance were entered as independent variables (fixed factors).

The rationale for this analysis is that the range of the direct medical cost of different diseases may be overlapped. By this way, factors that accounted for the variation in the cost could be identified.

In the second model, studies reporting costs of hospitalization, outpatient care and medications were extracted separately. Likewise, raw direct medical cost was selected as the dependent variable; all the other characteristic variables extracted from individual studies that were the same in model one together with categorized costs of hospitalisation, outpatient care and medications were entered as independent variables (fixed factors). In this way, the latter three variables that were linearly correlate with the total cost were de-linearized.

It is worth to note that GDP/Capita was entered into both models with the aim to testify that if it was correlated with the direct medical cost. The significant correlation between these two variable may support the later conversion of raw cost data into percentage of GDP/Capita.

Meta-analysis

For the meta-analysis, the converted direct medical cost was input as the individual effect size (ES). Both parametric (Random effects) ([Lipsey and Wilson 2000](#_ENREF_3)) and Non-parametric (Bootstrapping) methods ([Adams, Gurevitch et al. 1997](#_ENREF_2)) were adopted to compute the mean and 95% confidence intervals (CI) of the weighted ES ([Zhao, Xie et al. 2013](#_ENREF_4)). Since random-effects model accounts for the random effects variance, which represents the variability across the population effects, the biggest difference between fixed-effects and random-effects models is the significance levels (effects that were significant under a fixed-effects model may no longer be significant) and confidence intervals (confidence intervals will get bigger). Furthermore, if sample size is highly related to ES, then the mean ES will differ between the two models. So the random-effects model will generate more conservative result comparing to fixed-effects model and the fixed-effects model was not adopted in our analysis.

The equations used to calculate ES are following:

$$\overline{ES}=\frac{\sum(w\times ES)}{\sum w}$$

$$Q=\sum(w\times ES^{2})-\frac{\left[\sum\left(w\times ES\right)\right]^{2}}{\sum w}$$

$$\hat{v}_{\theta}=\frac{Q_{T}-k-1}{\sum w-\left(\frac{\sum w^{2}}{\sum w}\right)}$$

$w$ weight; $se$ standard error; k total number of studies; ES effect size

Reference: Lipsey MW, Wilson DB (2000) Practical Meta-Analysis SAGE Publications, Inc.

For the bootstrapping analysis, a random number of studies with replacement were chosen and then a weighted mean effect size was calculated in the bootstrapping method. This process was repeated for another 1999 times, the output values were rank-ordered sequentially, and the lowest and highest 2.5% values chosen as the bootstrap confidence limits.

In the parametric meta-analysis, the inverse variance is a better approach to weight the effect size of each study. However, for the COI studies included in this systematic review, almost all of them did not provide the variance of the cost (probably as the cost data is highly skewed). Therefore, in this case, a simple approach that uses sample size of individual study was adopted to give weight to each study as suggested by a statistic book ([Lipsey and Wilson 2000](#_ENREF_3)). Moreover, this approach has been employed in a published study investigating the transferability of indirect cost of illness studies earlier ([Zhao, Xie et al. 2013](#_ENREF_4)).

*Dealing with missing data in the meta-analysis*

For missing values on sample size in the studies, they were replaced by the average of remaining studies for that particular variable within a disease subgroup for the meta-analysis. Specifically, 8 (16.7%) studies of T2DM group, 3 (14.3%) studies of SC group, and 3 (8.1%) studies of EP group had the missing values on sample size.

In addition, in order to avoid putting unbalanced weight on study with extreme sample size, sample size for studies with greater than 10000 subjects were replaced with the mean sample size of the remaining studies following the method reported in study by Zhao et al. Therefore, 16, 7 and 4 studies from T2DM, SC and EP disease groups were removed before computing, respectively.

Data were analyzed using SPSS 20.0 (SPSS Inc. Chicago, IL, USA) and Microsoft Excel.

*Heterogeneities across cost of illness studies*

I^2^ value was computed to quantify the heterogeneities across inclusive studies via the following formula:

*I^2^=100%×(Q-df)/Q*

Where Q is Cochrane’s heterogeneity statistic and df is the degree of freedom. Negative values of I^2^ are put equal to zero so that I^2^ lies between 0% and 100%. A value of 0% indicates no observed heterogeneity, and larger values show increasing heterogeneity (according to the Cochrane handbook for Cochrane reviews).

- 0% to 40%: might not be important;
- 30% to 60%: may represent moderate heterogeneity*;
- 50% to 90%: may represent substantial heterogeneity*;
- 75% to 100%: considerable heterogeneity*.

*The importance of the observed value of I^2^ depends on (i) magnitude and direction of effects and (ii) strength of evidence for heterogeneity (e.g. P value from the chi-squared test, or a confidence interval for I^2^).

**References**

"The World Bank. Available at: <http://data.worldbank.org/indicator/NY.GDP.PCAP.CD> Accessed 6th June, 2013."

Adams, D. C., et al. (1997). "Resampling tests for meta-analysis of ecological data." Ecology **78**(4): 1277-1283.

Lipsey, M. W. and D. B. Wilson (2000). Practical Meta-Analysis SAGE Publications, Inc.

Zhao, F. L., et al. (2013). "Transferability of Indirect Cost of Chronic Disease: A Systematic Review and Meta-Analysis." Pharmacoeconomics **31**: 501-8.
