# Supplementary material for: Can the Direct Medical Cost of Chronic Disease Be Transferred across Different Countries? Using Cost-of-Illness Studies on Type 2 Diabetes, Epilepsy and Schizophrenia as Examples
Source: PLoS One. 2016 Jan 27;11(1):e0147169. doi: 10.1371/journal.pone.0147169 (PMC4731392; doi:10.1371/journal.pone.0147169)
Supplement: S1 Table — (DOCX) [file pone.0147169.s003.docx]

S 1 Table. Characteristics of included studies (2011 USD)

| Groups | | Country/Region | Conducting year | Data source | Sample size | Methods | Longitu  dinal | Age | Female | Direct cost | GDP/Cap | Percentage | Inpatient cost (%) | Outpatient cost (%) | Drug cost  (%) | Tests cost (%) |
| --- | --- | --- | --- | --- | --- | --- | --- | --- | --- | --- | --- | --- | --- | --- | --- | --- |
| DM | 1 | African Region^1^ | 2005 | Published | 974000 | modelling, prevalence | 0 | na | na | 902 | 12046 | 0.07488 | 0.14946 | 0.19246 | 0.555 | 0.103095 |
| DM | 2 | African Region^1^ | 2005 | Published | 536000 | modelling, prevalence | 0 | na | na | 984 | 4331 | 0.22720 | 0.155767 | 0.162395 | 0.581529 | 0.10031 |
| DM | 3 | African Region^1^ | 2005 | Published | 5510000 | modelling, prevalence | 0 | na | na | 1228 | 1054 | 1.16509 | 0.020577 | 0.017663 | 0.79467 | 0.16718 |
| DM | 4 | Brazil^2^ | 2007 | Survey | 1000 | bottom-up, prevalence | 0 | 59 | 67 | 1100 | 12594 | 0.08734 | na | na | 0.482 | na |
| DM | 5 | Canada | 1998 | Database | 1296048 | top-down, prevalence | 0 | classified | 51 | 2797 | 50565 | 0.05531 | 0.50209 | 0.19185 | 0.30605 | na |
| DM | 6 | China | 2007 | Survey | 2054 | bottom-up, prevalence | 0 | 62 | 67 | 1496 | 5439 | 0.27505 | na | na | na | na |
| DM | 7 | China | 2011 | Survey | 9396 | bottom-up, prevalence | 0 | 52 | 46 | 863 | 5439 | 0.15867 | 0.68896 | 0.048495 | 0.2592 | na |
| DM | 8 | Colombia | 2007 | Published | na | modelling, prevalence | 0 | na | na | 312 | 7100 | 0.04394 | na | na | na | na |
| DM | 9 | Germany | 1998 | Survey | 809 | bottom-up, prevalence | 0 | 66 | na | 4048 | 43865 | 0.09228 | 0.53681 | 0.1502 | 0.2898 | na |
| DM | 10 | Germany | 2000 | Survey | 6437 | na | 0 | 12.5 | 48 | 3151 | 43865 | 0.07183 | 0.2602 | 0.0901 | 0.2101 | 0.3701 |
| DM | 11 | Germany | 1999 | Database | na | top-down, prevalence | 0 | na | na | 4384 | 43865 | 0.09994 | 0.3553 | 0.1478 | 0.1912 | na |
| DM | 12 | Germany | 2001 | Database | 26971 | top-down, prevalence | 0 | na | 53 | 5989 | 43865 | 0.13653 | 0.357415 | 0.16667 | 0.19118 | ana |
| DM | 13 | Germany | 2007 | Database | na | top-down, prevalence | 0 | na | na | 8510 | 43865 | 0.19400 | na | na | na | na |
| DM | 14 | Hong Kong | 2004 | Survey | 147 | bottom-up, prevalence | 0 | na | na | 1777 | 34162 | 0.05202 | na | na | na | na |
| DM | 15 | India^3^ | 2005 | Survey | 309 | bottom-up, prevalence | 0 | 56.2 | 37.2 | 261 | 1528 | 0.17081 | na | na | na | na |
| DM | 16 | India^3^ | 2005 | Survey | 247 | bottom-up, prevalence | 0 | 54.8 | 40.5 | 164 | 1528 | 0.10733 | na | na | na | na |
| DM | 17 | India | 2009 | Survey | 718 | bottom-up, prevalence | 0 | 56 | 47.3 | 542 | 1528 | 0.35471 | 0.5092 | 0.1144 | 0.3395 | na |
| DM | 18 | Iran | 2005 | Survey | 710 | bottom-up, prevalence | 1 | 53.25 | 55 | 175 | 6799 | 0.02574 | 0.2857 | 0.1486 | 0.2857 | 0.206 |
| DM | 19 | Iran | 2009 | Survey | na | bottom-up, prevalence | 0 | classified | 56 | 883 | 6799 | 0.12987 | 0.0951 | 0.06 | 0.2378 | 0.09 |
| DM | 20 | Israel | 2001 | Database | 24632 | top-down, prevalence | 0 | Classified | 52.4 | 3304 | 32123 | 0.10285 | 0.3901 | 0.21 | 0.29 | na |
| DM | 21 | Italy^2^ | 1998 | Survey | 1668 | bottom-up, prevalence | 0 | na | na | 864 | 36124 | 0.02392 | na | 0.17 | 0.13 | 0.42 |
| DM | 22 | Italy | 1998 | Survey | 1263 | bottom-up, prevalence | 0 | na | na | 4601 | 36124 | 0.12737 | na | na | na | na |
| DM | 23 | Italy | 2002 | Database | 33792 | top-down, prevalence | 0 | classified | 49 | 5062 | 36124 | 0.14013 | 0.5719 | 0.1189 | 0.2531 | na |
| DM | 24 | Korea | 2005 | Survey | na | bottom-up, prevalence | 0 | na | na | 8055 | 23067 | 0.34920 | na | na | na | na |
| DM | 25 | Latin America | 2000 | Published | 339035 | modelling, prevalence | 0 | na | na | 918 | 10063 | 0.09123 | 0.1166 | 0.1743 | 0.3878 | na |
| DM | 26 | Norway | 2005 | Database | na | top-down, prevalence | 0 | na | na | 8858 | 98565 | 0.08987 | 0.09397 | 0.11196 | 0.42491 | na |
| DM | 27 | Pakistan^2^ | 2006 | Survey | 345 | bottom-up, prevalence | 0 | classified | 37 | 220 | 1182 | 0.18613 | 0.2447 | 0.0846 | 0.4591 | na |
| DM | 28 | Spain^4^ | 2002 | Database | 1842835 | top-down, prevalence | 0 | na | na | 2050 | 31820 | 0.06442 | 0.589 | 0.2177 | 0.1966 | na |
| DM | 29 | Spain^4^ | 2002 | Database | 1842835 | top-down, prevalence | 0 | na | na | 2277 | 31820 | 0.07156 | 0.5301 | 0.1754 | 0.17694 | na |
| DM | 30 | Spain^4^ | 2002 | Database | 1842835 | top-down, prevalence | 0 | na | na | 2153 | 31820 | 0.06766 | 0.5607 | 0.1964 | 0.18716 | na |
| DM | 31 | Sweden | 1998 | Survey | 777 | bottom-up, prevalence | 0 | na | na | 4268 | 57134 | 0.07470 | 0.4242 | 0.3089 | 0.2668 | na |
| DM | 32 | Sweden | 1993 | Survey | 1677 | top-down, prevalence | 0 | classified | na | 3222 | 57134 | 0.05639 | 0.3122 | 0.1024 | 0.198 | na |
| DM | 33 | Switzerland | 1999 | Survey | 1479 | bottom-up, prevalence | 0 | 66 | 50 | 3344 | 85794 | 0.03898 | 0.529 | 0.1687 | 0.3017 | na |
| DM | 34 | Thailand | 2001 | Database | 186 | bottom-up, prevalence | 0 | 62 | 62 | 201 | 5318 | 0.03780 | 0.1592 | 0.2388 | 0.4478 | na |
| DM | 35 | Thailand | 2008 | Survey | 475 | bottom-up, prevalence | 0 | 59 | 74 | 209 | 5318 | 0.03930 | 0.4928 | 0.1292 | 0.1435 | na |
| DM | 36 | United Kingdom | 2000 | Survey | 701 | bottom-up, prevalence | 0 | 64.3 | 43 | 2905 | 38918 | 0.07464 | 0.5002 | 0.2699 | 0.2499 | na |
| DM | 37 | United Kingdom^5^ | 2011 | Database | 3,800,000 | top-down, prevalence | 0 | na | na | 4020 | 38918 | 0.10329 | 0.3384 | na | 0.6053 | na |
| DM | 38 | USA | 1992 | Database | 471000 | top-down, prevalence | 0 | na | na | 5548 | 48328 | 0.11480 | na | na | na | na |
| DM | 39 | USA | 2002 | Database | 12100000 | modelling, prevalence | 0 | na | na | 16558 | 48328 | 0.34262 | 0.5902 | 0.2175 | 0.1907 | na |
| DM | 40 | USA^6^ | 2003 | Database | 6618 | top-down, prevalence | 0 | 52 | 45 | 4138 | 48328 | 0.08562 | 0.1725 | 0.1575 | 0.5498 | 0.1202 |
| DM | 41 | USA^6^ | 2003 | Database | 2441 | top-down, prevalence | 0 | 54.55 | 38.8 | 12775 | 48328 | 0.26434 | 0.4386 | 0.1656 | 0.2901 | 0.1039 |
| DM | 42 | USA^7^ | 2005 | Database | 21592 | top-down, prevalence | 0 | 51.7 | 48 | 14161 | 48328 | 0.29302 | 0.3065 | 0.4249 | 0.2686 | na |
| DM | 43 | USA^7^ | 2005 | Database | 127254 | top-down, prevalence | 0 | 51.7 | 48 | 14225 | 48328 | 0.29434 | 0.2393 | 0.4516 | 0.3091 | na |
| DM | 44 | USA^8^ | 2006 | Survey | 913 | bottom-up, prevalence | 0 | classified | 46 | 20568 | 48328 | 0.42559 | na | na | na | na |
| DM | 45 | USA^8^ | 2006 | Survey | 3320 | bottom-up, prevalence | 0 | classified | 53.1 | 9715 | 48328 | 0.20102 | na | na | na | na |
| DM | 46 | USA | 2004 | Survey | 8429 | bottom-up, prevalence | 0 | 60 | 52 | 5049 | 48328 | 0.10447 | na | na | na | na |
| DM | 47 | USA | 2007 | Published | na | modelling, prevalence | 0 | na | na | 10498 | 48328 | 0.21722 | na | na | na | na |
| DM | 48 | USA | 2012 | Database | na | modelling, prevalence | 0 | na | na | 13422 | 48328 | 0.27773 | 0.2077 | 0.2055 | 0.5868 | na |
| SC | 1 | Australia | 2000 | Database | 980 | bottom-up, prevalence | 0 | na | na | 27856 | 67039 | 0.41552 | 0.7792 | 0.0332 | 0.0499 | na |
| SC | 2 | Australia | 2003 | Survey | 347 | bottom-up, prevalence | 1 | 33.8 | 38 | 12223 | 67039 | 0.18233 | 0.42 | 0.0578 | 0.0801 | na |
| SC | 3 | Canada | 1996 | Published | 221000 | top-down, prevalence | 0 | na | 50 | 7172 | 50565 | 0.14184 | na | na | na | na |
| SC | 4 | Canada | 2004 | Published | 234305 | top-down, prevalence | 0 | Classified | 49 | 8332 | 50565 | 0.16478 | 0.3763 | 0.0712 | 0.0745 | na |
| SC | 5 | France | 1992 | Survey | 477 | bottom-up, prevalence | 0 | na | na | 17114 | 42642 | 0.40134 | 0.548 | 0.095 | 0.056 | na |
| SC | 6 | India^2^ | 2005 | Survey | 50 | bottom-up, prevalence | 0 | 32.9 | 38 | 151 | 1528 | 0.09882 | na | 0.0397 | 0.4768 | na |
| SC | 7 | Italy | 1995 | Survey | 99 | bottom-up, prevalence | 0 | classified | 50 | 14530 | 36124 | 0.40223 | 0.165 | 0.0578 | 0.07 | na |
| SC | 8 | Italy | 1998 | Survey | 553 | bottom-up, prevalence | 1 | classified | 38 | 33269 | 36124 | 0.92097 | 0.1993 | 0.1055 | 0.1145 | na |
| SC | 9 | Korea | 2005 | Database | 161058 | top-down, prevalence | 0 | classified | 44 | 29942 | 23067 | 1.29804 | 0.607 | 0.144 | 0.025 | na |
| SC | 10 | Nigeria^9^ | 1997 | Survey | 50 | bottom-up, prevalence | 0 | 42.9 | na | 101 | 1509 | 0.06693 | na | na | na | na |
| SC | 11 | Spain | 2001 | Survey | na | bottom-up, prevalence | 1 | na | na | 4788 | 31820 | 0.15047 | na | na | na | na |
| SC | 12 | Spain | 2001 | Survey | na | bottom-up, prevalence | 1 | na | na | 4913 | 31820 | 0.15440 | na | na | na | na |
| SC | 13 | Spain | 2001 | Survey | na | bottom-up, prevalence | 1 | na | na | 6298 | 31820 | 0.19793 | na | na | na | na |
| SC | 14 | Taiwan | 2003 | Survey | 46 | bottom-up, prevalence | 0 | 33.8 | 43 | 4016 | 21900 | 0.18338 | na | na | na | na |
| SC | 15 | Taiwan | 1999 | Database | 52432 | top-down, prevalence | 0 | 39.7 | 55 | 2895 | 21900 | 0.13219 | 0.2547 | 0.2015 | 0.2565 | na |
| SC | 16 | Taiwan | 2008 | Survey | 74 | bottom-up, prevalence | 0 | 43 | 33.7 | 2815 | 21900 | 0.12854 | 0.2973 | 0.0614 | 0.4481 | na |
| SC | 17 | Thailand | 2008 | Survey | 3557692 | bottom-up, prevalence | 0 | na | na | 1059 | 5318 | 0.19914 | 0.5 | na | na | na |
| SC | 18 | United Kingdom | 1997 | Published | 7500 | modelling, incidence | 0 | na | na | 27284 | 38918 | 0.70106 | 0.6407 | 0.26 | 0.02 | na |
| SC | 19 | England^10^ | 2005 | Survey | 122347 | bottom-up, prevalence | 0 | na | na | 33627 | 38918 | 0.86405 | 0.7924 | na | na | na |
| SC | 20 | USA | 2002 | Database | 15164 | top-down, prevalence | 0 | na | na | 19336 | 47882 | 0.40383 | 0.12 | 0.21 | 0.22 | na |
| SC | 21 | USA | 2007 | Database | 3966 | top-down, prevalence | 0 | na | 39 | 16804 | 47882 | 0.35095 | 0.3976 | 0.2542 | 0.3482 | na |
| EP | 1 | Hong Kong | 1996 | Survey | 745 | bottom-up, prevalence | 0 | na | na | 3782 | 34259 | 0.11039 | 0.353 | 0.39 | 0.238 | 0.019038 |
| EP | 2 | United Kingdom^11^ | 1990 | Database | 1628 | bottom-up, prevalence | 0 | na | na | 2260 | 38918 | 0.05807 | 0.592307 | 0.1846 | 0.2005 | na |
| EP | 3 | United Kingdom^11^ | 1990 | Database | 602 | bottom-up, incidence | 1 | na | na | 446 | 38918 | 0.01146 | na | na | na | na |
| EP | 4 | Spain^2^ | 2005 | Database | 762 | bottom-up, prevalence | 0 | 40.5 | 49.2 | 7725 | 32077 | 0.24083 | 0.2813 | 0.0637 | 0.5874 | 0.06861 |
| EP | 5 | Oman | 2000 | Survey | 486 | bottom-up, prevalence | 1 | classified | 50.2 | 1871 | 23572 | 0.07937 | 0.5179 | 0.1577 | 0.225 | 0.09781 |
| EP | 6 | Italy | 2004 | Survey | 631 | bottom-up, prevalence | 1 | classified | 49.6 | 1606 | 36267 | 0.04428 | 0.2254 | 0.1034 | 0.6034 | 0.06849 |
| EP | 7 | India | 1998 | Survey | 285 | bottom-up, prevalence | 0 | 22.6 | 44.9 | 106 | 1514 | 0.07001 | 0.1038 | 0.1038 | 0.6981 | 0.08491 |
| EP | 8 | USA | 2005 | Database | 4323 | top-down, prevalence | 0 | 42.7 | 56.7 | 11815 | 48328 | 0.24448 | 0.2841 | 0.3402 | 0.2744 | 0.03013 |
| EP | 9 | USA | 1995 | Database | 899 | top-down, incidence | 0 | na | na | 1582 | 48328 | 0.03273 | 0.2953 | 0.1233 | 0.3143 | 0.1517 |
| EP | 10 | Denmark | 2006 | Database | 64587 | top-down, prevalence | 0 | 42 | 48 | 4578 | 59709 | 0.07667 | 0.692 | 0.078 | 0.2302 | 0.05242 |
| EP | 11 | China | 2008 | Survey | 289 | bottom-up, prevalence | 0 | classified | 54.6 | 389 | 5417 | 0.07181 | 0.0925 | 0.0257 | 0.653 | 0.11825 |
| EP | 12 | Germany | 2003 | Survey | 101 | bottom-up, prevalence | 1 | 40.7 | 53.5 | 5117 | 44111 | 0.11600 | 0.2771 | 0.0096 | 0.5925 | 0.02013 |
| EP | 13 | USA | 2004 | Survey | 2837 | bottom-up, prevalence | 0 | 48.1 | 55.5 | 5387 | 48328 | 0.11147 | 0.2953 | 0.1233 | 0.3143 | 0.04455 |
| EP | 14 | Sweden | 2009 | Published | 53674 | top-down, prevalence | 0 | classified | classified | 2011 | 57638 | 0.03489 | 0.3153 | 0.275 | 0.4112 | 0.119344 |
| EP | 15 | Mexico | 2004 | Survey | 72 | bottom-up, prevalence | 0 | 28.8 | 58.3 | 3151 | 10146 | 0.31057 | 0.2101 | 0.3875 | 0.2412 | 0.160901 |
| EP | 16 | Italy | 2000 | Survey | 525 | bottom-up, prevalence | 1 | classified | 54 | 2158 | 36267 | 0.05950 | 0.2595 | 0.0709 | 0.6696 | 0.11121 |
| EP | 17 | USA^13^ | 1994 | Published | 335167 | modelling, prevalence | 0 | na | na | 4231 | 48328 | 0.08755 | 0.5542 | 0.1233 | 0.1718 | 0.200425 |
| EP | 18 | USA^13^ | 1994 | Published | 335167 | modelling, incidence | 0 | na | na | 6247 | 48328 | 0.12926 | 0.4800 ^12^ | na | 0.1138 | na |
| EP | 19 | France, Germany, UK | 1993 | Survey | 300 | bottom-up, prevalence | 0 | 35.4 | 45 | 4234 | 44007 | 0.09621 | 0.4192 | 0.1294 | 0.3368 | 0.076523 |
| EP | 20 | United Kingdom | 1993 | Database | 785 | bottom-up, prevalence | 0 | na | na | 1589 | 38918 | 0.04083 | 0.584 | 0.0761 | 0.2329 | 0.058274 |
| EP | 21 | France | 1998 | Survey | 1942 | bottom-up, incidence | 1 | classified | classified | 3356 | 44007 | 0.07626 | 0.6797 | 0.053 | 0.0805 | 0.186233 |
| EP | 22 | USA | 1994 | Survey | 192 | modelling, incidence | 0 | na | na | 12775 | 48328 | 0.26434 | 0.2953 | 0.1233 | 0.3143 | 0.018787 |
| EP | 23 | Spain | 2010 | Survey | 171 | bottom-up, prevalence | 1 | 41.6 | 47.7 | 1566 | 32077 | 0.04882 | 0.2953 | 0.1233 | 0.3143 | 0.15326 |
| EP | 24 | USA | 1996 | Published | 9090 | top-down, prevalence | 0 | 38 | 53.4 | 13787 | 48328 | 0.28528 | 0.5342 | 0.346 | 0.0925 | 0.01255 |
| EP | 25 | Nigeria | 2010 | Survey | 69 | bottom-up, prevalence | 0 | 35 | 52 | 208 | 1522 | 0.13666 | 0.2953 | 0.1233 | 0.6827 | 0.110577 |
| EP | 26 | China | 2011 | Survey | 500 | bottom-up, prevalence | 0 | 35.3 | 41 | 594 | 5417 | 0.10965 | 0.1886 | 0.1233 | 0.4815 | 0.082492 |
| EP | 27 | USA^14^ | 1995 | Published | na | bottom-up, prevalence | 0 | na | na | 2199 | 48328 | 0.04550 | 0.515 | na | 0.244 | 0.207 |
| EP | 28 | USA^14^ | 1995 | Published | na | top-down, prevalence | 0 | na | na | 2229 | 48328 | 0.04612 | 0.4212 | 0.2601 | 0.2299 | 0.07223 |
| EP | 29 | Netherland | 1999 | Survey | 116 | bottom-up, prevalence | 0 | 44 | 49.1 | 3409 | 50216 | 0.06789 | 0.2793 | 0.1757 | 0.2995 | 0.154884 |
| EP | 30 | India | 2001 | Survey | 182 | bottom-up, prevalence | 0 | na | na | 80 | 1514 | 0.05284 | 0.1125 | 0.3125 | 0.4375 | 0.0375 |
| EP | 31 | United Kingdom | 1991 | Survey | 303 | top-down, prevalence | 0 | 37 | 48 | 1673 | 38918 | 0.04299 | 0.1727 | 0.2367 | 0.4866 | 0.109384 |
| EP | 32 | USA | 2009 | Published | 1026 | top-down, prevalence | 0 | classified | 50.16 | 9073 | 48328 | 0.18774 | 0.2953 | 0.1233 | 0.3143 | 0.026452 |
| EP | 33 | Burundi | 2001 | Survey | 352 | bottom-up, prevalence | 0 | 26.3 | 42.2 | 2 | 275 | 0.00727 | 0.22 | 0.065 | 0.5 | na |
| EP | 34 | Italy | 1996 | Survey | 2307 | top-down, prevalence | 0 | 25 | 50.8 | 1826 | 36267 | 0.05035 | 0.7459 | 0.0542 | 0.1336 | 0.06736 |
| EP | 35 | Germany | 2008 | Database | 366 | bottom-up, prevalence | 0 | 59.5 | 45.9 | 3249 | 44111 | 0.07366 | 0.3321 | 0.0471 | 0.2669 | 0.138812 |
| EP | 36 | USA | 2000 | Published | na | modelling, incidence | 0 | classified | classified | 13304 | 48328 | 0.27529 | classified | classified | classified | classified |
| EP | 37 | USA | 2003 | Database | 500 | top-down, incidence | 0 | classified | 54.4 | 4010 | 48328 | 0.08297 | 0.43 | 0.173 | 0.061 | 0.312 |

Notes: prospectively: 0: No; 1: Yes. na: not available; classified: data was presented as categorical variable. DM: type 2 diabetes mellitus; SC: schizophrenia; EP: epilepsy

Footnote:

^1^ international dollar was used in the value of 2005.

^2^ Inpatient costs were not included.

^3^ costs were reported for urban and rural areas separately in the same study.

^4^ costs were based on three different prevalence (5%, 5.5% and 6.0%)

^5^ costs for primary care were presented.

^6^ based on the same study, one-estimation was for T2DM patient without major cardiovascular disease (MCV) and the other is for patient with MCV.

^7^ costs were for 2000 and 2005 respectively.

^8^ based on the same study, one was for patient with MCV, the other was not.

^9^ Inpatient cost included only.

^10^ presented results were based on institutional sample.

^11^ results were from one study with both prevalent and incident costs.

^12^ this figure represents both inpatient and outpatient costs.

^13^ calculations were for prevalent and incident cases respectively.

^14^ study adopted both bottom-up and top-down methods, thus the results were presented separately
